# Increased regional body fat is associated with depressive symptoms: a cross-sectional analysis of NHANES data obtained during 2011–2018

**DOI:** 10.1186/s12888-024-05782-4

**Published:** 2024-05-03

**Authors:** GuiMei Zhang, Sisi Wang, Ping Ma, Shuna Li, Xizhe Sun, Yang Zhao, Jiyang Pan

**Affiliations:** 1 Department of Psychiatry, Sleep Medicine Centre, First Affiliated Hospital of Jinan University, Guangzhou, Guangdong Province 510632 P.R. China; 2 Department of Clinical Research, The First Affiliated Hospital of Jinan University, Guangzhou, Guangdong 510632 China

**Keywords:** Adults, Cross-sectional study, Depressive symptoms, Fat mass, NHANES

## Abstract

**Aims:**

The findings from previous epidemiological studies of the association between regional body fat and depressive symptoms have been unclear. We aimed to determine the association between the body fat in different regions and depressive symptoms based on data from the National Health and Nutrition Examination Survey (NHANES).

**Methods:**

This study included 3393 participants aged ≥ 20 years from the NHANES performed during 2011–2018. Depressive symptoms were assessed using the Patient Health Questionnaire-9. The fat mass (FM) was measured in different regions using dual-energy X-ray absorptiometry to determine the total FM, trunk FM, arm FM, and leg FM. The FM index (FMI) was obtained by dividing the FM in kilograms by the square of the body height in meters. Weighted data were calculated in accordance with analytical guidelines. Linear logistic regression models were used to quantify the association between regional FMI and depressive symptoms. Univariate and stratified analyses were also performed.

**Results:**

The participants in this study comprised 2066 males and 1327 females. There were 404 (11.91%) participants with depressive symptoms, who were aged 40.89 ± 11.74 years and had a body mass index of 30.07 ± 7.82 kg/m². A significant association was found between total FMI and depressive symptoms. In the fully adjusted multivariate regression model, a higher total FMI (odds ratio = 2.18, 95% confidence interval [CI] = 1.08–4.39) was related to a higher risk of depressive symptoms, while increased total FMI (β = 1.55, 95% CI = 0.65–2.44, *p* = 0.001), trunk FMI (β = 0.57, 95% CI = 0.04–1.10, *p* = 0.036), and arm FMI (β = 0.96, 95% CI = 0.33–1.59, *p* = 0.004) were significantly associated with PHQ-9 (Patient Health Questionnaire-9) scores, whereas the leg FMI was not (*p* = 0.102). The weighted association between total FMI and depressive symptoms did not differ significantly between most of the subpopulations (all *p* values for interaction > 0.05). The risk of having depression was higher in individuals who were non-Hispanic Whites, smokers, drinkers, obese, and had diabetes and thyroid problems (*p* < 0.05).

**Conclusion:**

These findings suggest that the population with a higher regional FMI is more likely to have depressive symptoms, especially in those who also have an increased total FMI. The association is more pronounced in individuals who are smokers, drinkers, obese, and have diabetes and thyroid problems.

**Supplementary Information:**

The online version contains supplementary material available at 10.1186/s12888-024-05782-4.

## Introduction

Obesity is one of the main public health problems worldwide, and it also increases the risks of chronic diseases such as depression, metabolic diseases, cardiovascular diseases, musculoskeletal disorders, Alzheimer’s disease, and certain types of cancer, and leads reductions in the quality of life and life expectancy [1]. The World Health Organization reported that 39% of adults older than 18 years were overweight in 2018 and that 13% were obese in 2016. In most countries worldwide, more people die from being overweight and obese than from being underweight, and the global prevalence of obesity nearly tripled between 1975 and 2016 [2]. Overweight and obesity have become a public health problem affecting the health of most of the world population.

Depression is one of the most prevalent mental health conditions worldwide, with a global prevalence of approximately 4.7% and an annual incidence rate of 3.0% [3]. It was recognized as a key contributor to disability by the Global Burden of Disease Study [4] and is frequently linked with unhealthy lifestyle habits [5–7], inadequate physical activity [8], and elevated body mass index (BMI) [9]. Additionally, a significant proportion of this population experiences subclinical depressive symptoms that impact their quality of life and functional abilities [10]. Between 2010 and 2018, the number of adults in the US diagnosed with depression increased by 12.9%, from 15.5 million to 17.5 million. During this period the additional economic cost on adults with depression surged by 37.9%, from USD 236.6 billion to USD 326.2 billion (adjusted to 2020 values). Projections by the World Health Organization suggest that depression will represent 13% of global diseases by 2030, surpassing cardiovascular conditions as the most-burdensome ailment [11].

The possible clinical and epidemiological associations between depression and obesity have been investigated since the early 1960s [12], but they remain controversial. For example, Konttinen et al. and Silva et al. suggested that there is a positive relationship between BMI and depressive symptoms [13–15], but no other studies have produced similar findings [16, 17]. A U-shaped relationship between increased rates of depressive symptoms and being underweight and obese was also found recently [18–20], **i**ncluding in a study that analyzed data from the National Health and Nutrition Examination Survey (NHANES) [21]. A meta-analysis of longitudinal studies found that changes in BMI were associated with a reduction in depression [22]. Even though the findings have not been consistent, recent studies have found that adipocytes (e.g., leptin and adiponectin among white adipose tissue) link obesity, depression [23], and bipolar disorder as a biological factor [24].

BMI is now a widely used indicator for measuring and categorizing obesity. It is used to assess the development or prevalence of risk factors for several health problems and is widely used when formulating public health policies. However, since the human body includes various constituents such as muscle, fat, bone, and water, individuals with the same BMI can have markedly different body compositions [25]. BMI might therefore be inadequate for accurately assessing the relationship between depression and fat tissue because it does not accurately reflect the distribution of body fat (e.g., subcutaneous and visceral fat), or for evaluating metabolic disorders in the body [26].

The fat mass index (FMI) has recently been suggested to be a more accurate indicator of body fat than BMI, since it is independent of other body constituents such as lean mass. A relationship between regional body fat and depression has not been reported previously, and so we aimed to characterize this relationship by analyzing the NHANES data obtained during 2011–2018 in order to improve public awareness of the health effects of body fat.

## Methods

### Study design

**Fig. 1:**
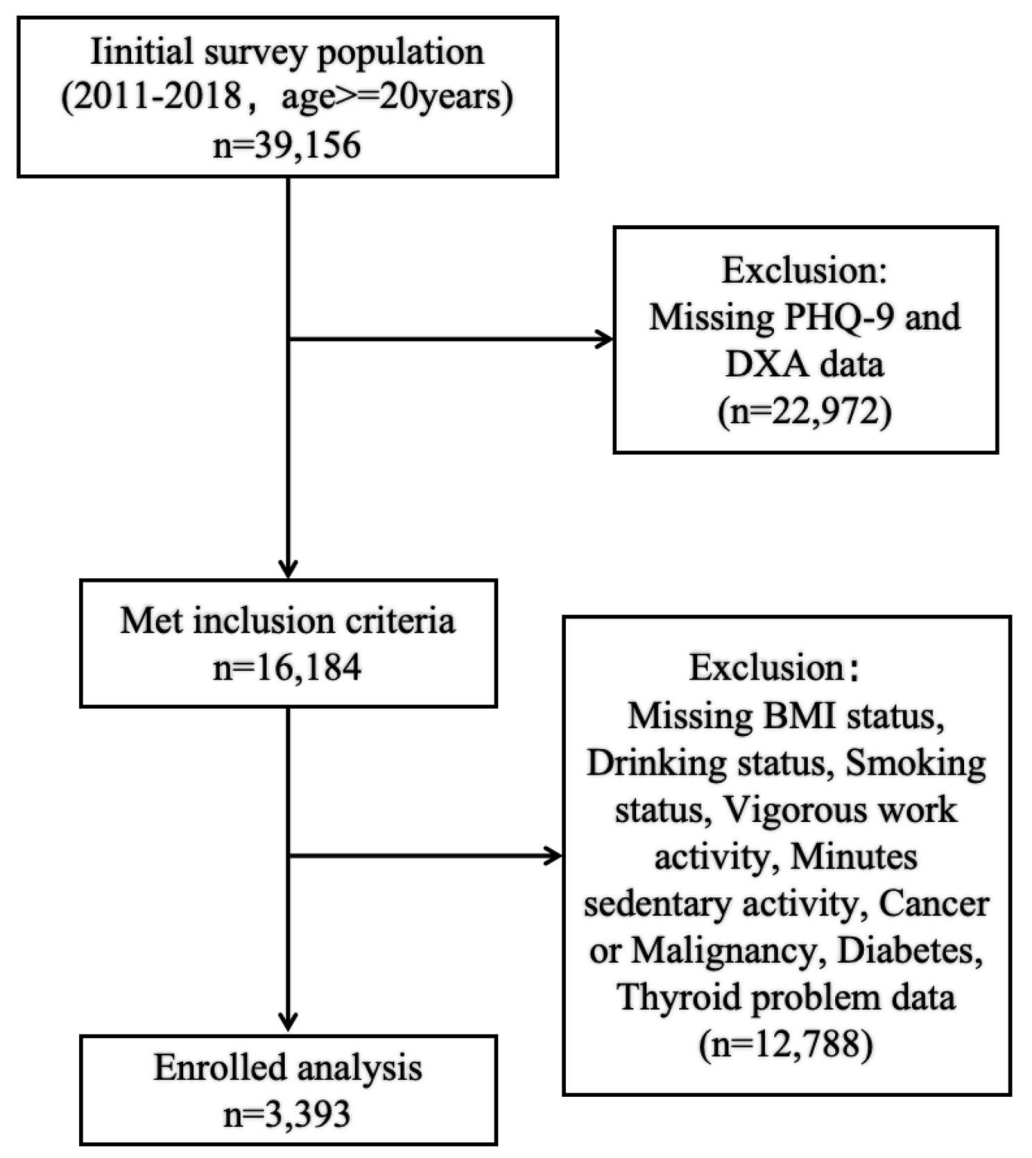
Flow chart of participants included

The NHANES is a US national stratified multistage probability sampling program performed by the National Center for Health Statistics (NCHS) to assess nutritional status and its associations with health promotion and disease prevention. The present analysis of data obtained in the NHANES performed during 2011–2018 initially identified 39,156 potential subjects for inclusion. Rigorous screening yielded 3393 participants aged ≥ 20 years with a complete set of data for Patient Health Questionnaire-9 (PHQ-9) scores, regional FMI values, and covariates, who were finally included in this study. The detailed screening process for participant inclusion is shown in Fig. 1. The Institutional Review Board of the NCHS approved the study protocol, and all participants signed consent forms.

### Data collection and definitions

#### Depressive symptoms

Depressive symptoms was assessed using PHQ-9 [27], which is a self-reported depressive symptoms screening tool based on the nine items that reflect major depressive disorder in the Fifth Edition of the Diagnostic and Statistical Manual of Mental Disorders. PHQ-9 is a reliable and valid measure of the severity of depressive symptoms that asks questions about the frequency of depressive symptoms experienced over the previous 2 weeks. Each question is scored from 0 (not at all) to 3 (nearly every day), with the final questionnaire score ranging from 0 to 27. Scores of at least 10 were considered to indicate depressive symptoms [28].

#### Fat mass index

The FMI was determined by dividing the fat mass (FM) in kilograms by the square of the body height in meters. Professional operators performed whole-body DXA scans of the participants, with quality control and other analyses used to produce measurements of soft tissue and bone in various regions of the body. The analysis included measurements of total FM, FM of the left and right arms, FM of the left and right legs, and trunk FM. The FM of the arms and legs was calculated as the sum of the FM of the limbs on each side. The following exclusion criteria were applied for the DXA scans: pregnancy, self-reported use of radiographic contrast (barium) within the previous 7 days, self-reported weight exceeding 450 pounds, or height over 6’5”. The scanning was performed using a densitometer (Discovery model A, Hologic, Bedford, MA, USA).

#### Covariates

We assessed the sociodemographic and lifestyle factors associated with regional FMI and depressive symptoms, including sex, age, race, education level, smoking status, drinking status, BMI, total cholesterol, high-density lipoprotein, sedentary time, physical activity, and comorbid diseases. The current smoking status was categorized into smoking or nonsmoking based on the question “Do you smoke now?” Similarly, the drinking status was categorized into drinking or nondrinking based on the question “Do you drink alcohol now?” BMI was calculated by trained health technicians at the Mobile Examination Center (MEC) as the weight in kilograms divided by the square of the body height in meters. Obesity was defined as a BMI of ≥ 30 kg/m^2^. Total cholesterol and high-density lipoprotein were measured using standardized methods. Sedentary time was quantified in minutes per day and determined by the response to the question “How much time do you usually spend sitting on a typical day?” Physical activity was categorized into the three intensity levels of light, moderate, and vigorous based on the answers to questions such as “Do you engage in any vigorous/moderate-intensity activities in a typical week?” and “Do you participate in any vigorous/moderate-intensity recreational activities in a typical week?” Chronic diseases were assessed through the self-reported medical history, and included diabetes, cancer/malignancy, and thyroid problems. Participants were asked whether a doctor had informed them of having any of these conditions.

### Statistical analysis

All of the interviews and MEC examination weights included in this study can be found in the detailed demographic files that can be accessed at the following website: https://wwwn.cdc.gov/nchs/nhanes/tutorials/module3.aspx. Since the individuals examined by the MEC were a subset of those interviewed in the survey, we combined the MEC examination weights in the analysis. The data were weighted based on guidance provided by NCHS analysts on combining multiple cycles and constructing appropriate weights. The associations between regional FMI and depressive symptoms were examined using multivariate logistic regression models. Three models were constructed: Model 1 was adjusted for age, sex, and race; Model 2 was adjusted for age, race, sex, education level, physical activity, and sedentary activity; and Model 3 was adjusted for age, race, sex, education level, physical activity, sedentary activity, BMI, drinking status, smoking status, cancer/malignancy, diabetes, thyroid problems, total cholesterol, and high-density lipoprotein. Additionally, linear regression models were used to describe the association between regional FMI and PHQ-9 scores with the same three sets of adjusted models described above. The effect values were expressed as odds ratios (ORs), β coefficients, and corresponding 95% confidence intervals (CIs). Stratified analyses with subgroup variables were conducted using Model 3. Univariate and stratified analyses were performed to identify independent effects between total FMI and depressive symptoms. An interaction test was also conducted on this factor, adjusted using Model 3. The weighted-sample independent *t*-test or chi-square test was employed to analyze baseline information in the control and depression groups. Continuous variables conforming to a normal distribution were presented as mean ± standard-deviation values, while numbers and weighted percentages were used for categorical variables. All statistical analyses were performed using the R software (version 3.6.1, https://www.r-project.org/). A two-sided probability value of *p* < 0.05 was considered statistically significant in all analyses.

## Results

### Weighted participant characteristics

The participants in this study comprised 2066 males and 1327 females. There were 404 (11.91%) participants with depressive symptoms, who were aged 40.89 ± 11.74 years and had a BMI of 30.07 ± 7.82 kg/m^2^. The total, trunk, arm, and leg regional FMIs were higher in those with depressive symptoms, at 1.49 ± 0.63, 2.26 ± 1.01, 1.67 ± 0.80, and 2.97 ± 1.31 kg/m^2^, respectively (all *p* < 0.001). The weighted baseline characteristics according to depressive symptoms are listed in Table 1. Those with depressive symptoms were more likely to be female, smokers, drinkers, and have a higher education level and BMI, and less likely to have diabetes or thyroid problems (all *p* < 0.05). Depressive symptoms were significantly associated with BMI, sex, education level, smoking status, drinking status, diabetes, physical activity, and thyroid problems (all *p* < 0.05), but not with age, race, cancer/malignancy, total cholesterol, or high-density lipoprotein (all *p* > 0.05) (Table 1).

**Table 1:** Weighted baseline characteristics

Variables		Depression (*n* = 3393)		*P*
		No (*n* = 2989)	Yes (*n* = 404)	
**Age (years)**		40.61 ± 11.55	40.89 ± 11.74	0.711
**BMI status (kg/ m** ^**2**^ **)**		28.38 ± 6.23	30.07 ± 7.82	0.001^*^
**Gender(%)**	Male	1907 (61.10)	159 (39.45)	< 0.001^*^
	Female	1082 (38.90)	245 (60.55)	
**Race (%)**	Mexican American	412 (9.18)	34 (6.37)	0.088
	Other Hispanic	264 (5.86)	49 (7.16)	
	Non-Hispanic White	1346 (69.14)	200 (66.40)	
	Non-Hispanic Black	532 (8.34)	75 (10.27)	
	Other Race	432 (7.50)	46 (9.80)	
**Education level (%)**	Less than 9th grade	156 (3.25)	25 (4.85)	0.013^*^
	9-11th grade	486 (12.71)	85 (17.39)	
	High school graduate/GED or equivalent	804 (27.43)	113 (29.48)	
	Some college or AA degree	1009 (34.62)	144 (35.94)	
	College graduate or above	534 (22.00)	37 (12.34)	
**Smoking status (%)**	Yes	1261 (37.27)	237 (60.50)	< 0.001^*^
**Drinking status (%)**	Yes	738 (24.23)	152 (39.14)	< 0.001^*^
**Diabetes (%)**	Yes	45 (6.91)	17 (12.81)	0.001^*^
**Thyroid problem (%)**	Yes	174 (6.98)	61 (14.88)	< 0.001^*^
**Cancer or Malignancy (%)**	Yes	134 (6.39)	30 (6.65)	< 0.866
**Physical activity (%)**	vigorous	1501 (52.08)	149 (37.17)	< 0.001^*^
	moderate	838 (29.31)	114 (27.10)	
	light	650 (18.60)	141 (35.72)	
**Total FMI (kg/ m** ^**2**^ **)**		1.25 ± 0.50	1.49 ± 0.63	< 0.001^*^
**Trunk FMI (kg/ m** ^**2**^ **)**		1.92 ± 0.83	2.26 ± 1.01	< 0.001^*^
**Arm FMI (kg/ m** ^**2**^ **)**		1.36 ± 0.62	1.67 ± 0.80	< 0.001^*^
**Leg FMI (kg/ m** ^**2**^ **)**		2.45 ± 1.08	2.97 ± 1.31	< 0.001^*^
**Sedentary activity (min/day)**		374.91 **±** 203.41	422.92 **±** 225.45	0.007^*^
**Total cholesterol (md/dL)**		195.03 **±** 43.70	194.14 **±** 41.15	0.060
**High-density lipoprotein (md/dL)**		52.09 **±** 16.40	50.06 **±** 15.76	0.717

Continuous variables conforming to a normal distribution were presented as mean and standard-deviation values, while numbers and weighted percentages were used for categorical variables. All estimates were weighted to be nationally representative

^*^ indicates statistically significant difference (*P* < 0.05)

### Weighted associations between different regional FMIs and depressive symptoms in the entire study population

**Fig. 2:**
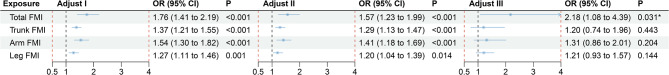
Weighted association between the regional fat mass index (FMI) and depressive symptoms based on logistic regression models Data are presented as odds ratios (ORs), 95% confidence intervals (CIs), and *p* values Model 1 was adjusted for age, sex, and race Model 2 was adjusted for age, sex, race, education level, physical activity, and sedentary activity Model 3 was adjusted for age, race, sex, education level, physical activity, sedentary activity, body mass index (BMI), drinking status, smoking status, cancer/malignancy, diabetes, thyroid problems, total cholesterol, and high-density lipoprotein

Figure 2 presents the associations between regional FMIs and depressive symptoms in the logistic regression models. Overall, the ORs for having depressive symptoms relative to not having them were higher in individuals with higher total, trunk, arm, and leg FMIs, at 1.76 (95% CI = 1.41–2.19), 1.37 (95% CI = 1.21–1.55), 1.54 (95% CI = 1.30–1.82), and 1.27 (95% CI = 1.11–1.46) (*p* < 0.05), respectively, in Model 1, and 1.57 (95% CI = 1.23–1.99), 1.29 (95% CI = 1.13–1.47), 1.41 (95% CI = 1.18–1.69), and 1.20 (95% CI = 1.04–1.39) (*p* < 0.05) in Model 2. In Model 3 the significant association persisted for the total FMI (OR = 2.18, 95% CI = 1.08–4.39) (*p* = 0.031), but not for the trunk, arm, or leg FMI (*p* > 0.05).

### Weighted associations between different regional FMIs and PHQ-9 scores in the entire study population

**Fig. 3:**
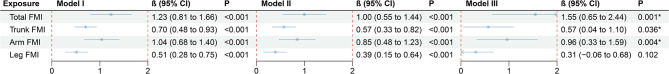
Weighted association between the regional FMI and PHQ-9 (Patient Health Questionnaire-9) scores in linear regression models Data are presented as β values, 95% CIs, and *p* values

Compared with participants without depressive symptoms, in Model 1 the total FMI (β = 1.23, 95% CI = 0.81–1.66), trunk FMI (β = 0.70, 95% CI = 0.48–0.93), arm FMI (β = 1.286, 95% CI = 0.68–1.40), and leg FMI (β = 0.51, 95% CI = 0.28–0.75) were positively associated with PHQ-9 scores (*p* < 0.001). These positive associations were still present after making the extra adjustments in Model 2 (all *p* < 0.001) (Fig. 3). In Model 3, increased total FMI (β = 1.55, 95% CI = 0.65–2.44, *p* = 0.001), trunk FMI (β = 0.57, 95% CI = 0.04–1.10, *p* = 0.036), and arm FMI (β = 0.96, 95% CI = 0.33–1.59, *p* = 0.004) were still significantly related to PHQ-9 scores, whereas the leg FMI was not (*p* = 0.102) (Fig. 3).

### Weighted effect size of total FMI on depressive symptoms in prespecified and exploratory subgroups based on logistic regression models

**Fig. 4:**
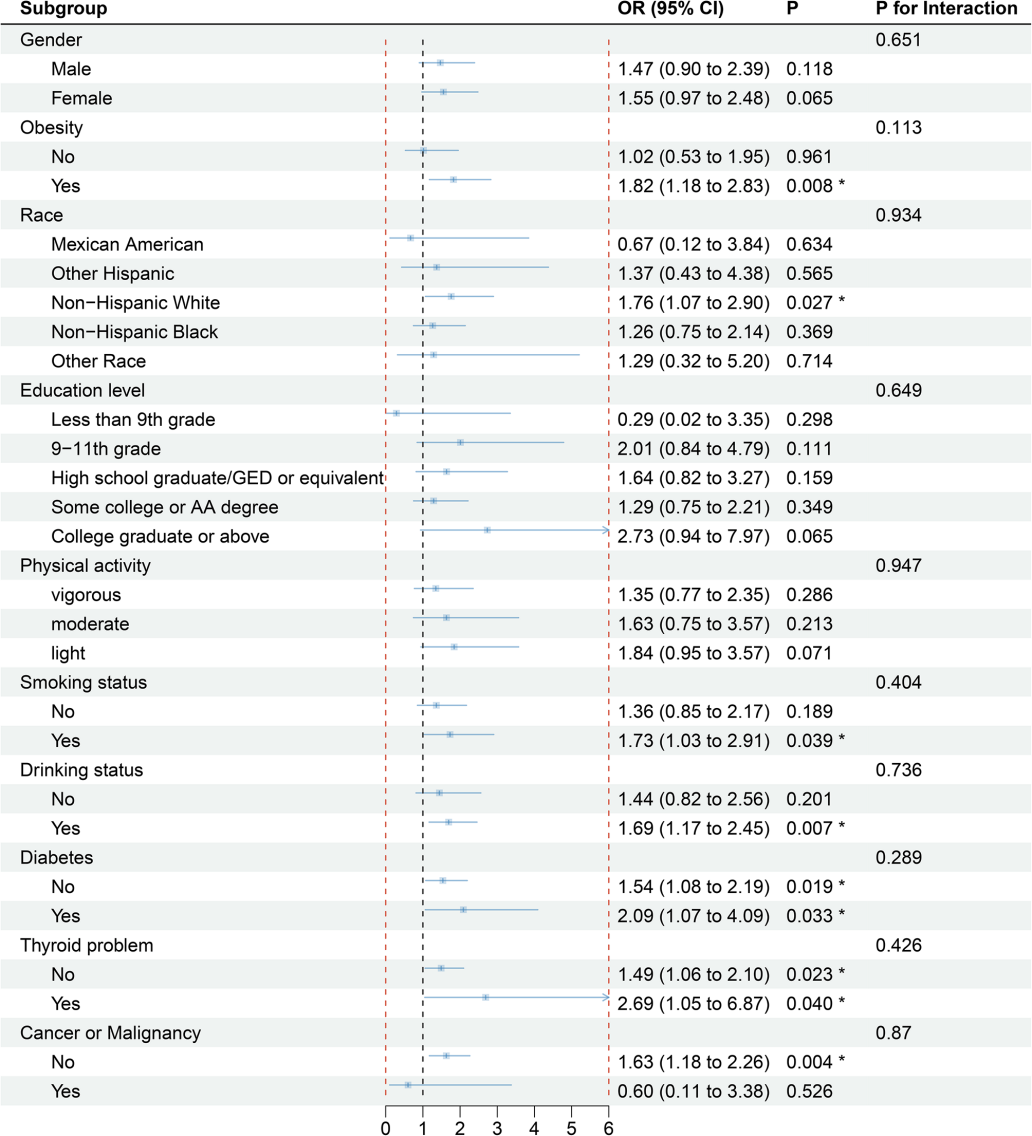
Forest plot showing the weighted effect sizes (ORs) of total FMI on depressive symptoms in prespecified and exploratory subgroups based on logistic regression Each stratification was adjusted for all factors (age, race, sex, education level, physical activity, sedentary activity, BMI, drinking status, smoking status, cancer/malignancy, diabetes, thyroid problems, total cholesterol, and high-density lipoprotein) except the stratification factor itself. *: *p* < 0.05

Subgroup analyses were performed to estimate the robustness of the associations of depressive symptoms with total FMI. In the fully adjusted multivariate model (except the stratification factor itself), the association between total FMI and depressive symptoms did not differ significantly between most of the subpopulations (all *p* values for interaction > 0.05) (Fig. 4). The ORs for having depressive symptoms along with elevated total FMI were higher in individuals who were non-Hispanic Whites (1.76, 95% CI = 1.07–2.90), smokers (1.73, 95% CI = 1.03–2.91), drinkers (1.69, 95% CI = 1.17–2.45), obese (1.82, 95% CI = 1.18–2.83) (*p* < 0.05), and had diabetes (2.69, 95% CI = 1.05–6.87) and thyroid problems (2.69, 95% CI = 1.05–6.87) (*p* < 0.05).

## Discussion

This national representative cross-sectional study found a positive association between regional body fat and depressive symptoms. A higher total FMI was significantly associated with depressive symptoms, and this association remained across various subgroups. Additionally, all of the analyzed regional FMIs other than the leg FMI were related to PHQ-9 scores. Finally, we observed a more-pronounced relationship between total FMI and depressive symptoms in individuals who were smokers, drinkers, obese, and had diabetes or thyroid problems.

To the best of our knowledge, this is the first study to directly investigate the association between the body fat in different regions and depressive symptoms, which distinguishes it from previous studies that have mostly focused on the relationship between BMI and depressive symptoms. As one of the most convenient clinical weight measurements, BMI ≥ 30 kg/m^2^ has widely been considered to represent obesity. However, BMI represents the sum of FMI and fat-free mass index [29], and the latter accounts for skeletal muscle mass, bone, and organs, while FMI accounts for peripheral and visceral adipose tissues, and so it does not separate FM from other body constituents to accurately reflect the distribution of body fat (e.g., subcutaneous and visceral fat) and hence is also not suitable for evaluating metabolic disorders in the body [26]. Different components of the body that contribute to BMI, such as fat, muscle, bone, and water, all make different contributions to the health status [30], and so individuals with the same BMI may have markedly different amounts of body fat in different regions [31]. Adipose tissue is widely distributed in the human body and plays a significant role in regulating various aspects of human physiology [32], such as in systemic metabolism, energy balance, and immune homeostasis [33]. Obesity triggers a series of alterations in adipose tissue, including adipocyte hyperplasia and/or hypertrophy [34], inhibition of angiogenic responses [35], local hypoxia [36], an altered secretory signature, and alterations in the inflammatory and immune landscape of adipose tissue [36]. Indeed, abnormal metabolism and inflammatory dysregulation are also common characteristics of depression [37, 38].

Changes in the function or number of different subsets of immune cells in adipose tissue are strongly related to the development of obesity-related chronic inflammation [39]. Dramatic changes in adipocyte secretion mostly include the increased secretion of proinflammatory adipokines such as interleukin 6 (IL-6), IL-8, IL-1β, leptin, resistin, tumor necrosis factor α (TNF-α), monocyte chemotactic protein-1, plasminogen activator inhibitor-1, progranulin, chemerin, and colony-stimulating factor-1, and reduced levels of anti-inflammatory mediators such as IL-10 and adiponectin [33]. Patients with depression exhibit similar changes associated with immune system activation, such as increased IL-6, TNF-α, and C-reactive protein levels [40, 41]. Increased adipose tissue upregulates macrophage synthesis to cause a significant shift from an anti-inflammatory M2 state to a proinflammatory M1 phenotype [42, 43]. Macrophage polarization caused by lipopolysaccharide or interferon-α signaling have also been found to be one of the most important immune causes of depression [44, 45]. M1 macrophages ultimately activate multiple inflammatory pathways and induce the release of numerous proinflammatory cytokines [46].

Obesity is also associated with dysregulation of the hypothalamic–pituitary–adrenal (HPA)-axis function and impaired negative cortisol feedback [47], both of which have been found to contribute to depressive disorder [48]. The potential underlying mechanism involves the brain receiving information about peripheral inflammatory processes via multiple communication routes, such as the humoral and neuronal pathways [49], and then proinflammatory cytokines circulating through the vagus nerve crossing the blood–brain barrier to the brain and hypothalamus to further result in nuclear factor κB (NF-κB) activation in the microglial cells of the hypothalamus and consequently leading to hypothalamic inflammation and leptin resistance [50, 51]. Related findings indicate that high-fat diets increase the expression of proinflammatory cytokines and the activation of the proinflammatory transcription factor NF-κB in the hypothalamus [52]. Glucocorticoid (GC) receptors involved in metabolic regulation and the distribution of body fat [53] also show regional variations in density, with elevated concentrations in visceral adipose tissue [54] in obesity. Some association may remain with decreased GC receptors, malfunctions in negative-feedback regulation, and cortisol hypersecretion in patients with depression [55, 56].

An additional mechanism that could explain the association of FM with depression is the influence of obesity on cognitive function and brain structure [57, 58]. Dysregulation of the HPA axis may be an important contributor, which along with the subsequent hypersecretion of GCs is linked to impaired cognition dysfunction in depression [47, 59]. Moreover, HPA-axis hyperactivity has been linked to reductions in hippocampal volume [60]. Previous studies have also found that BMI, and especially central obesity [61], was inversely associated with the global brain volume [62], possibly due to metabolic abnormalities in the gray and white matter [63] and the loss of neurons [64] in individuals with obesity. Obesity-associated systemic inflammation has been identified as a risk factor for depression and for cognitive dysfunction in the elderly [50], but our study could not confirm this since the participants were aged 20–60 years. Further stratified analysis indicated that higher total and trunk FMIs were both more-significant factors for depressive symptoms in females than in males.

Sex differences in the distribution of body fat have been found in previous studies [65], with there being more body fat in females. Another study found that this was mainly due to differences in sex hormones between males and females [66]. Especially in central obesity, the negative implications may be greater in females than in males, which might give rise to multiple metabolic dysregulations that could be responsible for a depressed state [5] (Fig. 2). We also found that people with obesity are significantly more likely to develop depression and other stress-related mood disorders in the four regional fat groups, which was similar to the results of previous studies [67, 68] (Fig. 2). This suggests that a high BMI influences the associations between different regional FMIs and depression. The differences in the regional FMIs were more strongly associated with depression in the smoking group than in the nonsmoking group (Fig. 2), which could also help to explain the results of previous studies [69, 70]. However, more studies are needed to determine the specific mechanisms underlying how regional body fat, smoking, obesity, and sex interact with each other to influence the incidence of depression, while the association between different types of FMI and depressive symptoms was still consistent in the other subgroup analyses, as presented in Supplemental Figures S1–S4.

This study had significant strengths that should be noted. The present findings can support clinical work involving adults with depression, such as focusing on adjusting an elevated FM to the optimal status, especially in those with chronic diseases. Furthermore, this study was the first that we were aware of to investigate the association between depression and FM in different body regions in adults, our study utilized a multi-ethnic, diverse, nationally representative population, including a relatively large sample of individuals with depressive symptoms, the large population included in this study all statistical processes were weighted and followed NHANES guidelines to amalgamate multiple cycles and determine fitting weights for use in this study. Univariate and stratified analyses were also performed to identify independent effects between total FMI and depressive symptoms. Reliable conclusions can be drawn, even not employing multiple imputation for large missing data. Also, together the present findings combined with previous ones for BMI have effectively illustrated that fat is a clinical factor in depression and in depressive symptom of other somatic diseases, which provides some clues to the mechanisms underlying depression. We found that sex, obesity status, and smoking status significantly influenced the association between the amounts of body fat in different regions and depression, with the positive association between them being stronger in female, obese, and smoking individuals. It should be noted that these existing disparities may affect the health of other individuals who are persistently obese or smokers.

Our study also had several inevitable limitations. First, although the study sample was sufficiently large, causal associations between depression and regional fat could not be estimated due to the cross-sectional design. Second, DXA screening was only performed on young and middle-aged adults, and so the results could not be generalized to the elderly. Third, these findings may mostly only be applicable to the US and might not be directly extrapolatable to other race groups and regions. Notwithstanding these limitations, this was a valuable population-based study that focused on the association between depression and regional fat in adults. There should have more prospective studies focus on this relationship between depression and regional body fat, such as Cohort studies, Randomized Controlled Trials. Specifically, reasonable interventions are implemented in future research, such as healthy eating, exercise plans, and psychological support. And incorporating an untreated appropriate control group and assessing the relationship directly between actual regional body fat and improvement in depressive symptoms, utilizing more sophisticated statistical analysis to reduce biases, includes factors associated with regional body fat and mediators. Furthermore, long-term follow-up studies are necessary to observe the enduring effects of interventions on depressive symptoms and regional body fat, which helps ascertain the sustained impact of interventions and potential long-term health outcomes, evaluating whether these effects persist or diminish with regional body fat maintenance. Studies have reported significant positive relationships between weight loss and degree of depressive symptoms improvement in obesity population [71, 72]. So, it would be of value to see a more active exploration in the study of depressive benefits of regional body fat loss.

## Conclusions

This study found that regional body fat was independently associated with a higher risk of depressive symptoms in US adults, especially in those who also have increased total body fat. This association was present even after adjusting for case complexities, and was more pronounced in individuals who are smokers, drinkers, obese, and have diabetes and thyroid problems in the prespecified subgroups.

### Electronic supplementary material

Below is the link to the electronic supplementary material.

**Supplementary Material 1:** The legends for Supplementary Figures 1–4

Supplementary Material 2

## Data Availability

The datasets generated and analyzed for the current study are available in the NHANES repository. These data can be accessed using the following link: https://wwwn.cdc.gov/nchs/nhanes/Default.aspx.
