# Supplementary material for: Increased regional body fat is associated with depressive symptoms: a cross-sectional analysis of NHANES data obtained during 2011–2018
Source: BMC Psychiatry. 2024 May 3;24:336. doi: 10.1186/s12888-024-05782-4 (PMC11067210; doi:10.1186/s12888-024-05782-4)
Supplement: Supplementary file 1 — Supplementary Material 1: The legends for Supplementary Figures 1–4 [file 12888_2024_5782_MOESM1_ESM.docx]

Supplemental Figure 1. Forest plot showing the weighted effect sizes (β values) of total FMI on PHQ-9 (Patient Health Questionnaire-9) scores in prespecified and exploratory subgroups based on linear regression models. Each stratification was adjusted for all factors (age, race, sex, education level, physical activity, sedentary activity, BMI, drinking status, smoking status, cancer/malignancy, diabetes, thyroid problems, total cholesterol, and high-density lipoprotein) except the stratification factor itself. *: *p* < 0.05.

Supplemental Figure 2. Forest plot showing the weighted effect sizes (β values) of trunk FMI on PHQ-9 (Patient Health Questionnaire-9) scores in prespecified and exploratory subgroups based on linear regression models. Each stratification was adjusted for all factors (age, race, sex, education level, physical activity, sedentary activity, BMI, drinking status, smoking status, cancer/malignancy, diabetes, thyroid problems, total cholesterol, and high-density lipoprotein) except the stratification factor itself. *: *p* < 0.05.

Supplemental Figure 3. Forest plot showing the weighted effect sizes (β values) of arm FMI on PHQ-9 (Patient Health Questionnaire-9) scores in prespecified and exploratory subgroups based on linear regression models. Each stratification was adjusted for all factors (age, race, sex, education level, physical activity, sedentary activity, BMI, drinking status, smoking status, cancer/malignancy, diabetes, thyroid problems, total cholesterol, and high-density lipoprotein) except the stratification factor itself. *: *p* < 0.05.

Supplemental Figure 4. Forest plot showing the weighted effect sizes (β values) of leg FMI on PHQ-9 (Patient Health Questionnaire-9) scores in prespecified and exploratory subgroups based on linear regression models. Each stratification was adjusted for all factors (age, race, sex, education level, physical activity, sedentary activity, BMI, drinking status, smoking status, cancer/malignancy, diabetes, thyroid problems, total cholesterol, and high-density lipoprotein) except the stratification factor itself. *: *p* < 0.05.
