# Supplementary figures and images for: Increased regional body fat is associated with depressive symptoms: a cross-sectional analysis of NHANES data obtained during 2011–2018
Source: BMC Psychiatry. 2024 May 3;24:336. doi: 10.1186/s12888-024-05782-4 (PMC11067210; doi:10.1186/s12888-024-05782-4)

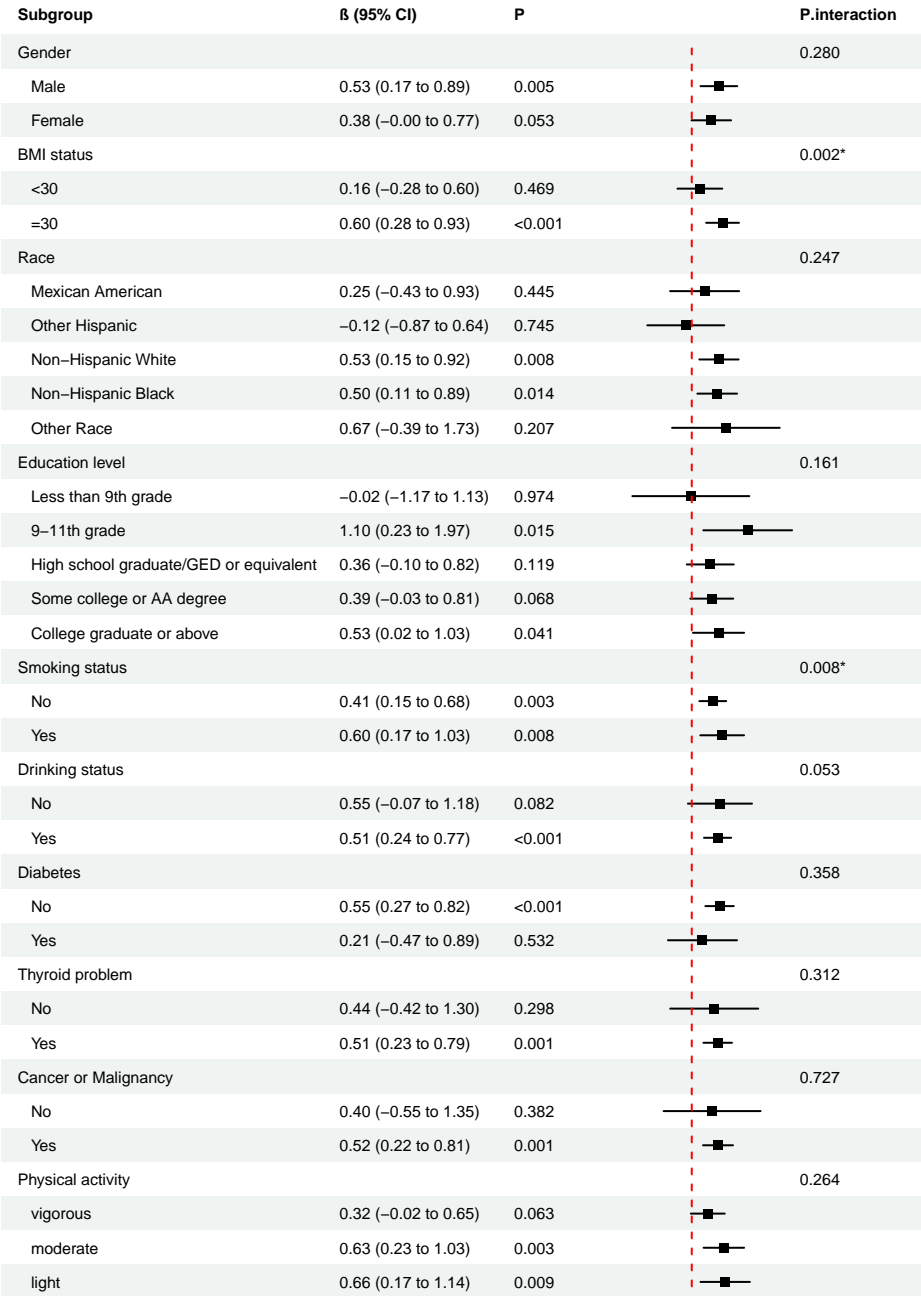

Effect size of Leg FMI on depression in prespecified and exploratory subgroups

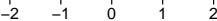

Supplement: Supplementary file 2 — Supplementary Material 2 [file 12888_2024_5782_MOESM2_ESM.zip › S4.LegFMIpdf.pdf]

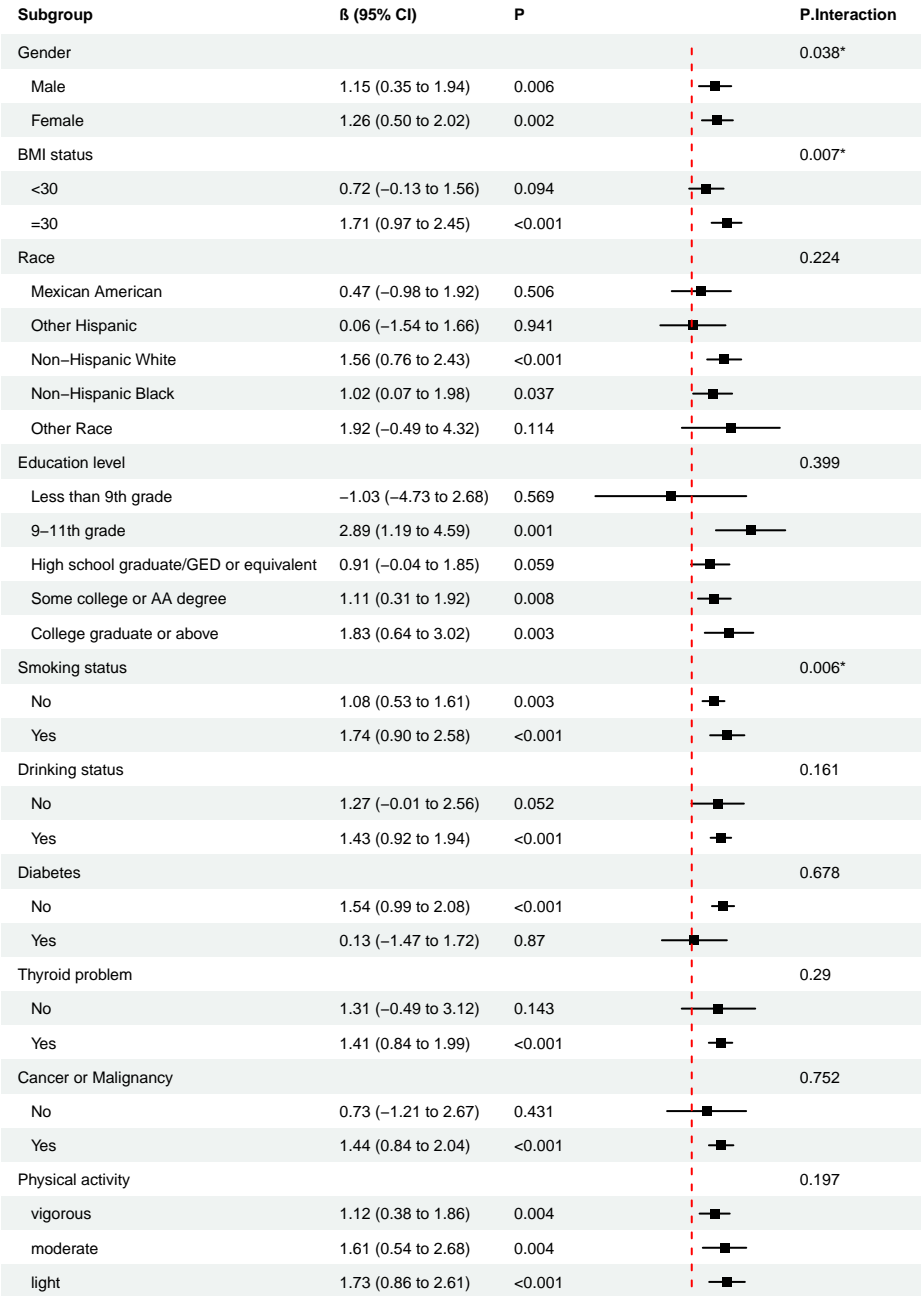

Effect size of Total FMI on depression in prespecified and exploratory subgroups

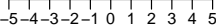

Supplement: Supplementary file 2 — Supplementary Material 2 [file 12888_2024_5782_MOESM2_ESM.zip › S1-TotalFMI.pdf]

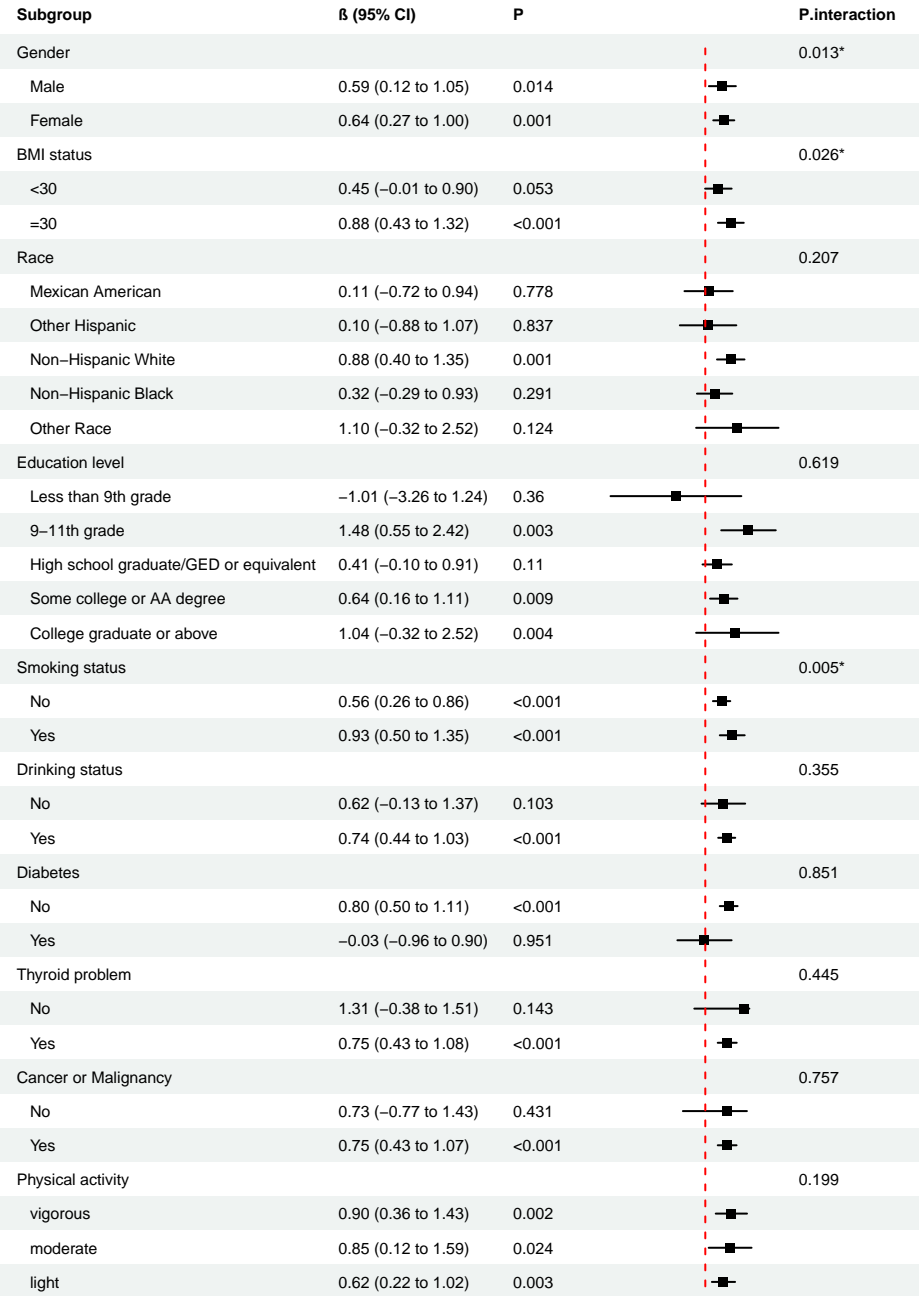

Effect size of Total FMI on depression in prespecified and exploratory subgroups

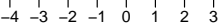

Supplement: Supplementary file 2 — Supplementary Material 2 [file 12888_2024_5782_MOESM2_ESM.zip › S2 TrunkFMI.pdf]

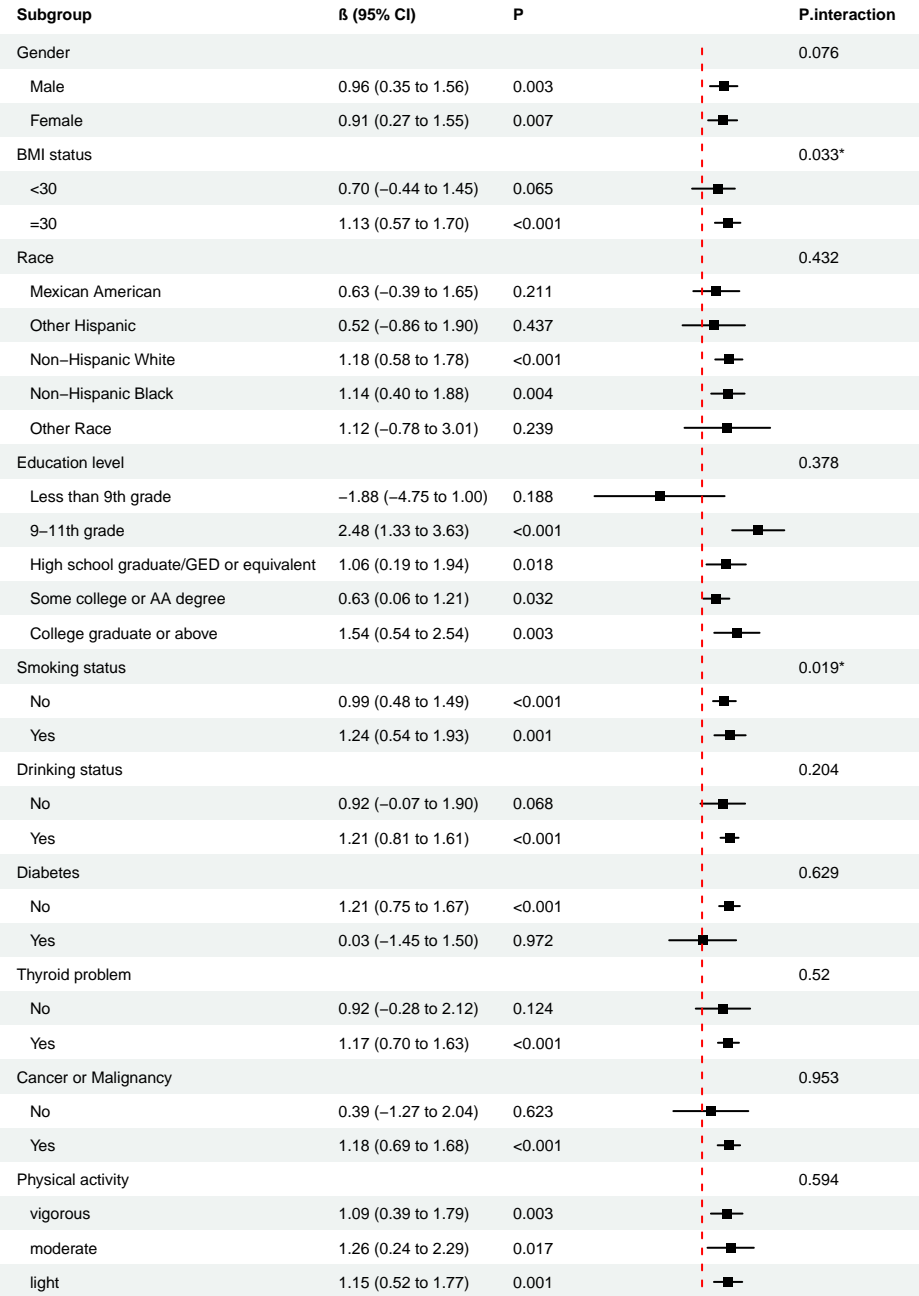

Effect size of Arm FMI on depression in prespecified and exploratory subgroups

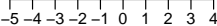

Supplement: Supplementary file 2 — Supplementary Material 2 [file 12888_2024_5782_MOESM2_ESM.zip › S3 ArmFMI.pdf]
